# Evaluating Lichen Sclerosus in Phimosis: Insights From a Multidisciplinary Retrospective Study

**DOI:** 10.1111/ajd.14417

**Published:** 2025-01-15

**Authors:** Stefano Bighetti, Stefano Mancon, Nazareno Suardi, Piergiacomo Calzavara‐Pinton, Vincenzo Maione, Mariachiara Arisi, Giovanni Lughezzani, Nicola Zerbinati, Iacopo Ghini, Luca Bettolini

**Affiliations:** ^1^ Dermatology Department University of Brescia, ASST Spedali Civili di Brescia Brescia Italy; ^2^ Department of Biomedical Sciences Humanitas University Milan Italy; ^3^ Department of Urology IRCCS Humanitas Research Hospital Milan Italy; ^4^ Urology Department University of Brescia, ASST Spedali Civili di Brescia Brescia Italy; ^5^ Dermatology Unit, Department of Medicine and Surgery University of Insubria, ASST Dei Sette Laghi Varese Italy; ^6^ Pathology Department University of Brescia, ASST Spedali Civili di Brescia Brescia Italy

**Keywords:** circumcision, dermatological consultation, lichen sclerosus, phimosis, preputial pathologies

## Abstract

**Objectives:**

This study aimed to evaluate the prevalence of lichen sclerosus (LS) in male patients undergoing circumcision for phimosis, emphasising the significance of a multidisciplinary approach in the early diagnosis and management of this condition.

**Methods:**

A retrospective analysis was performed on 841 male patients who underwent circumcision at a high‐volume medical centre between 2001 and 2023. Data were collected on clinical diagnoses made by both dermatologists and urologists, along with the corresponding histological findings. Logistic regression models were utilised to assess diagnostic accuracy and the relationship between clinical suspicion and histological confirmation.

**Results:**

Histological confirmation of LS was identified in 30.6% of the cases. Dermatologists demonstrated a diagnostic accuracy of 81.8%, whereas urologists showed an accuracy of 46.1%. The evaluations conducted by dermatologists exhibited a significant correlation with histological confirmation of LS (odds ratio 4.81; 95% confidence interval: 1.96–11.80; *p* = 0.001).

**Conclusions:**

This study elucidates the differences in diagnostic accuracy between dermatologists and urologists in identifying LS in patients undergoing circumcision for phimosis, highlighting the necessity of a multidisciplinary approach to improve the diagnostic process. Furthermore, the epidemiological data obtained from this research offer valuable insights into the prevalence of LS and the associated diagnostic challenges within a substantial patient cohort.

## 1 Introduction

Phimosis, characterised by the inability to retract the foreskin over the glans penis, represents a prevalent urological condition with diverse underlying aetiologies. Although congenital phimosis remains the most frequently observed form, acquired phimosis often arises secondary to chronic inflammatory conditions, notably lichen sclerosus (LS). LS is a chronic, progressive and inflammatory dermatosis that predominantly affects the anogenital region, with the most frequent localisation in men being the foreskin and glans, and is well‐documented as a significant cause of acquired phimosis [1, 2].

Diagnosing LS presents considerable challenges because of its subtle early symptoms, such as slight discoloration and thinning of the foreskin, which are often easily overlooked or misdiagnosed. The absence of timely and accurate diagnosis and management of LS can lead to complications, including not only phimosis but also meatal stenosis and an increased susceptibility to squamous cell carcinoma (SCC) [3, 4].

The intricacies of these potential outcomes underscore the critical need for heightened clinical awareness and a proactive and multidisciplinary management [5, 6].

This retrospective study was conducted to assess the prevalence of LS in a group of male patients undergoing circumcision for the treatment of phimosis. Furthermore, the study aimed to emphasise the importance of a collaborative, multidisciplinary approach in diagnosing and managing phimosis, by comparing the diagnostic accuracy of dermatologists and urologists in identifying LS, and highlighting the potential advantages of non‐surgical management for certain patients affected by LS.

## 2 Materials and Methods

### 2.1 Study Population

This retrospective analysis is based on data concerning male patients who underwent circumcision for phimosis at a single high‐volume centre from 2001 to 2023. The inclusion criteria for the study were male patients over the age of 14 with a diagnosis of phimosis treated surgically, where, at the time of the consultation leading to the indication for circumcision, a urologist or dermatologist had expressed a clinical suspicion of LS or of another underlying pathological condition causing phimosis. We identified 841 male patients who met the inclusion criteria of the study out of a total of 4831 circumcision procedures analysed. Every patient signed an informed consent form for data processing and the surgical procedure. The data extracted from the medical records included patient age and sex, pre‐existing diagnoses, details of prior medical treatments, circumcision date, primary indication for surgery (functional or suspected underlying mucocutaneous pathology), histological diagnosis, confirmation of LS or other identified mucocutaneous conditions, and records of prior dermatological and urological evaluations.

### 2.2 Statistical Analysis

Frequencies and percentages were reported for categorical variables, whereas medians and interquartile ranges (IQRs) were reported for continuous variables. Chi‐squared test was performed for categorical variables to compare the clinical suspicion groups regarding the histological diagnosis. Logistic regression analysis was fitted to investigate the correlation of clinical diagnosis with histological diagnosis. All tests were two‐sided. *p*‐values < 0.05 were considered indicative of statistical significance. Statistical analyses were conducted with Stata 17 (StataCorp LLC, College Station, TX, USA).

## 3 Results

A total of 841 male patients were selected for the study. The median age was 49 years (IQR 30–67). In 33 cases (3.92%), the clinical suspicion of LS was raised by dermatologists, in 102 cases (12.12%) by urologists, whereas in 706 cases (83.94%) circumcision for phimosis was performed in the absence of clinical suspicion of LS. Histological examination of tissue samples sent for histopathological analysis after circumcision revealed the presence of non‐specific posthitis in 341 cases (40.5%), LS in 257 cases (30.6%), lichenoid dermatitis in 158 cases (18.8%), lichen planus in 19 cases (2.3%), Zoon balanitis in 18 cases (2.1%), penile intraepithelial neoplasia(PeIN) or SCC in 16 cases (1.9%), condylomata in 5 cases (0.6%), psoriasis in 5 cases (0.6%), Paget's disease in 1 case (0.1%), Langerhans cell histiocytosis in 1 case (0.1%), lichenoid dermatitis and condylomata in 1 case (0.1%) and LS with concurrent presence of PeIN or SCC in 19 cases (2.3%) (Table 1). The diagnostic accuracy for LS was 46.1% for urologists, indicating that 46.1% of cases suspected by urologists were histologically confirmed as LS. In contrast, dermatologists achieved an accuracy of 81.8%, with 81.8% of their suspected LS cases being histologically confirmed.

**TABLE 1:** Main characteristics of the study population of 841 male patients treated with circumcision for phimosis.

Variable	Total, *n* (%)
Number of patients, *n* (%)	841
Age, median (IQR)	49 (30–67)
Histological diagnosis, *n* (%)	
Non‐specific posthitis	341 (40.5%)
LS	257 (30.6%)
Lichenoid dermatitis	158 (18.8%)
Lichen planus	19 (2.3%)
Zoon balanitis	18 (2.1%)
PeIN or SCC	16 (1.9%)
Condylomata	5 (0.6%)
Psoriasis	5 (0.6%)
Paget's disease	1 (0.1%)
Langerhans cell histiocytosis	1 (0.1%)
Lichenoid dermatitis/condylomata	1 (0.1%)
LS with concurrent PeIN or SCC	19 (2.3%)
Clinical suspicion of LS, *n* (%)	
None	706 (83.9%)
Urological	102 (12.1%)
Dermatological	33 (3.9%)

Abbreviations: IQR, interquartile range; LS, lichen sclerosus; PeIN, penile intraepithelial neoplasia; SCC, squamous cell carcinoma.

No statistically significant difference was detected between the urological and dermatological clinical suspicion groups in their correlation with the histological diagnosis of LS with a *p*‐value of 0.4. However, the logistic regression analysis revealed that clinical suspicion by dermatologists was associated with a significantly higher likelihood of histological confirmation of LS, with an odds ratio of 4.81 (95% CI: 1.96–11.80; *p* = 0.001) (Table 2).

**TABLE 2:** Logistic regression analysis investigating the correlation of urological and dermatological clinical suspicion of LS with histological diagnosis of LS.

	Clinical suspicion of LS	
	Urological	Dermatological
	OR; 95% CI; *p*‐value	OR; 95% CI; *p*‐value
Histological diagnosis of LS		
No	—	—
Yes	0.91; 0.60–1.38; 0.67	4.81; 1.96–11.80; 0.001

Abbreviations: CI, confidence intervals; LS, lichen sclerosus; OR, odds ratio.

## 4 Discussion

LS is a chronic inflammatory condition characterised by atrophic and scarring processes affecting the mucosa. Although research has predominantly focused on LS in female patients, diagnosing the condition presents challenges because of its varied clinical presentations.

Close collaboration between dermatologists and gynaecologists is essential for accurately diagnosing and effectively managing genital LS in female patients [7]. However, there is a relatively limited amount of information available regarding the diagnosis and management of genital LS in male patients.

Our study addresses this gap, demonstrating that dermatologists outperformed urologists in diagnostic accuracy, with rates of 81.8% and 46.1%, respectively. This may be attributed to dermatologists' use of advanced, non‐invasive diagnostic methods, such as dermoscopy, confocal microscopy and line‐field confocal optical coherence tomography, which enhance diagnostic capabilities across LS's wide clinical spectrum. Additionally, patients seeking urological consultation for phimosis often present with advanced stages of the condition, accompanied by considerable functional impairment or underlying balanopreputial pathologies that necessitate circumcision. In such cases, the primary emphasis of the urologist is on the surgical aspect rather than diagnostic procedures.

Of the preputial tissues analysed, 415 (49.2%) showed LS presence, with 257 (30.6%) in sclerotic‐atrophic form and 158 (18.8%) in active form (lichenoid dermatitis). The latter, representing 38.1% of LS cases, could potentially have benefited from early dermatological consultation and the initiation of topical therapies, such as corticosteroids, emollients and vitamin E: These treatments are cornerstones in the management of female LS, having shown efficacy in slowing, halting or reversing disease progression [7, 8]. Therefore, this subset of patients, affected by an active, non‐sclerotic LS form, and, in some instances, in more advanced cases, might have seen a significant benefit from early medical therapy, which could potentially delay or even obviate the need for surgical intervention by stabilising or regressing the pathological condition.

During the specified period, among the 4831 male patients aged 14 and above who were referred to our facility for circumcision, 11.6% presented with phimosis accompanied by underlying LS.

Reports in the literature of LS prevalence are highly variable, with histologically confirmed LS rates in circumcised adult males ranging from 1% to 67.4% [9]. This wide range reflects differences in patient populations, diagnostic criteria and clinical awareness of LS, underscoring the need for improved, standardised diagnostic approaches. Furthermore, these data offer valuable epidemiological insights into the role that LS might play in causing or contributing to the advancement of acquired phimosis.

Subjective symptoms can vary significantly among individual patients, often independent of the severity of the condition [9, 10]. In advanced stages of the disease, such symptoms may be linked to structural changes in the genital mucosa, which may require surgical intervention. However, in cases with subtle clinical presentations, particularly in early and non‐sclerotic/atrophic forms of LS, patients might delay seeking specialised medical attention, underestimating the seriousness of their condition. A delayed diagnosis poses a considerable challenge to therapeutic interventions, as early identification and treatment yield better responses to therapy and a lower risk of neoplastic transformation [7, 11]. However, only 3.9% of the patients in our cohort underwent dermatological evaluation before urological consultation for circumcision. This highlights a critical need to foster an interdisciplinary approach that comprises dermatological evaluation for patients with phimosis. Increasing routine referral to dermatologists could lead to earlier diagnosis, allowing for medical treatments that may prevent surgical intervention and contribute to better long‐term outcomes. Joint training programmes and shared clinical protocols between dermatologists and urologists could improve diagnostic precision and patient care. Furthermore, raising awareness among general practitioners and urologists about the benefits of a multidisciplinary approach could enhance the quality of life for male patients affected by LS, offering them a patient‐centred management strategy.

Although this study provides significant insights into understanding and managing LS in patients with phimosis, it is not without limitations. First and foremost, the retrospective design, although providing a broad overview of the treatment of this condition over a 20‐year period, inevitably introduces limitations related to data completeness and potential biases in patient selection. It is important to note that our study exclusively included patients who underwent circumcision, thus limiting the generalisability of the findings regarding non‐surgical therapeutic options, as patients who avoided surgical intervention were not considered. Moreover, the possibility that a portion of cases reported as “non‐specific posthitis” may represent an incipient or mild form of LS cannot be excluded. However, the extensive caseload and the long observation period provide a solid data foundation that highlights the potential of a multidisciplinary approach. A longitudinal approach could offer more insights into how early interventions affect disease progression and quality of life over time. Expanding the cohort to include patients who opted for conservative treatments could provide a more comprehensive picture of therapeutic modalities and their outcomes. The integration of non‐invasive diagnostic tools could also enrich our understanding of treatment response, leading to deeper discoveries that might influence the adoption of more targeted and personalised therapeutic approaches in the treatment of LS.

In conclusion, adopting a multidisciplinary approach with increased dermatological assessment for early diagnosis of LS could expand therapeutic alternatives, potentially delaying or avoiding surgery. Future prospective studies focusing on the efficacy of early medical intervention in preventing disease progression and improving patient outcomes would be valuable. Additionally, research into non‐invasive diagnostic tools and patient‐centred outcomes could further inform and optimise LS management strategies.

## Conflicts of Interest

The authors declare no conflicts of interest.

## Data Availability

The data that support the findings of this study are available from the corresponding author upon reasonable request.
